# High-Throughput Sequencing: A Roadmap Toward Community Ecology

**DOI:** 10.1002/ece3.508

**Published:** 2013-03-11

**Authors:** Timothée Poisot, Bérangère Péquin, Dominique Gravel

**Affiliations:** 1 Département de biologie, chimie et géographie, Université du Québec à Rimouski300 Allée des Ursulines, Rimouski, QC, G5L 3A1, Canada; 2 Québec Centre for Biodiversity Sciences, Stewart Biological Sciences Building1205 Dr. Penfield Avenue, Montréal, QC, H3A 1B1, Canada; 3 Département de Biologie, Université LavalQuébec, QC, G1V 0A6, Canada; 4 Québec-OcéanQuébec, QC, G1V 0A6, Canada; 5 Institut de Biologie Intégrative et des (IBIS) SystèmesQuébec, QC, G1V 0A6, Canada

**Keywords:** Biogeography, community ecology, high-throughput sequencing, microbial ecology

## Abstract

High-throughput sequencing is becoming increasingly important in microbial ecology, yet it is surprisingly under-used to generate or test biogeographic hypotheses. In this contribution, we highlight how adding these methods to the ecologist toolbox will allow the detection of new patterns, and will help our understanding of the structure and dynamics of diversity. Starting with a review of ecological questions that can be addressed, we move on to the technical and analytical issues that will benefit from an increased collaboration between different disciplines.

## Biogeography, microbes, and sequences

The new data created by joint advances in sequencing technologies and bioinformatics allowed a renaissance of microbial ecology and biogeography. Recent conceptual advances in metacommunity ecology (Leibold et al. 2004) allow recasting Baas Becking and Beijerinck's interrogation (De Wit and Bouvier 2006) of “is everything everywhere, and if not; does the environment select?” as a more integrative, mechanisms-focused inquiry. Microbial and community ecologists alike now seek to find the relative impact of neutral dynamics, dispersal limitations, and species sorting on the spatial distribution of different levels of diversity. Due to their short generation time, the different temporal and spatial scales at which they occur, and their presence in nearly all of Earth's environments, often along steep local environmental gradients, microbial communities make an ideal systems to investigate precise hypotheses formulated within such general questions (Green and Bohannan 2006). In addition, they have important functional diversity, being fundamental to the functioning of most ecosystems, and are easily manipulated (Buckling et al. 2009) or studied in nature (Weitz et al. 2013). For this reason, general ecologists can gain new information by paying more attention to these systems.

Tools allowing an accurate description of microbial communities are becoming available and accessible, and can be used to address outstanding hypotheses of biogeography (see, e.g., O'Dwyer and Green 2010), and further our understanding of how ecological communities assemble, evolve, and function. Currently, precise knowledge of the presence and absence of taxonomic or functional entities at several spatial scales is possible. Targeted tag pyrosequencing and other next-generation high-throughput sequencing (HTS) methods offer an unprecedented, cost-effective way to describe microbial biodiversity in a variety of systems and environments. These methods (called HTS henceforth; see Box 1 for a brief overview of the technologies) generate large quantities of nucleotide sequences, which translates into improved descriptions of diversity with a minimal amount of work and falling cost-per-sequence compared with earlier technologies (Tedersoo et al. 2010). In a nutshell, each sample is assigned a tag, that is, a unique identifier, added to the primer used for amplification; within this tag, sequences are individually read through various biochemical reactions (see references in Box 1). The output of this process is a list of sequences for each sample, which can be interpreted so as to represent taxonomic information, relative abundances, and other aspects of community structure, as we illustrate in this article.

**Box 1. A primer of high-throughput sequencing for ecologists**

**Figure 1:**
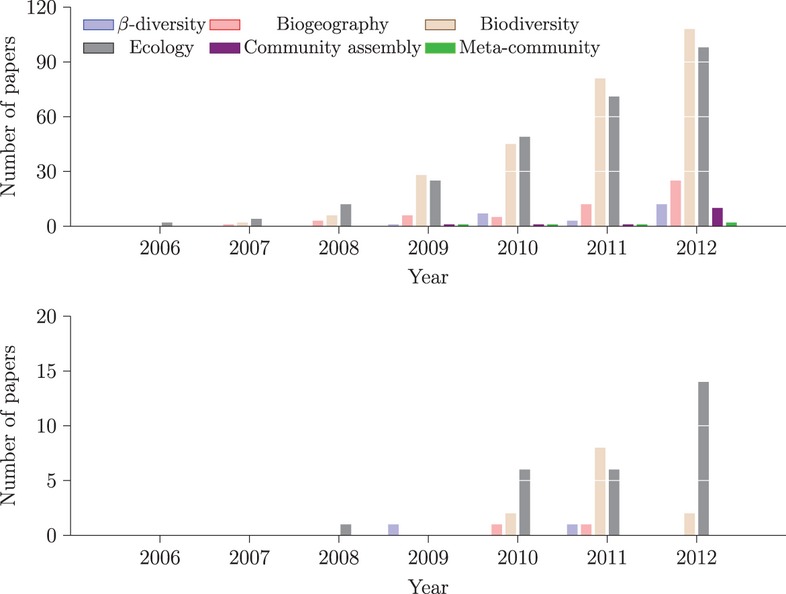
HTS technologies are being scarcely used in ecology, despite acceleration in the recent years. The top and bottom panel are, respectively, the number of hits for queries on six keywords (beta-diversity, biogeography, ecology, biodiversity, community assembly, and meta-community) with either pyrosequencing or Illumina, in Web of Science as of January 2013. There are two main conclusions to be drawn from this figure. First, 454 pyrosequencing is the most used technology in ecology. Second, specific topics have not been investigated yet, as attested by the lack of studies covering specific topics such as community assembly, or meta-community dynamics. It emphasizes that HTS should now be used to explore more focused hypotheses. Each interuption in the bars represents 30 papers in the top panel, and 5 papers in the bottom panel.

There are currently four main HTS platforms available, relying on different biochemical principles (Myllykangas et al. 2012) and tailored to suit different uses (Purdy and Hurd 2010). Two of them (PacBio and IonProton) are infrequently used in ecological studies. Rather, the dominant methods are Illumina GA-II and GS-FLX+ (454 pyrosequencing). GS-FLX+ produces less but longer sequences when compared with Illumina (on average, 1 million vs. billions of sequences, of length 400 vs. 150 basepairs). Due to these differences, Illumina is mostly used for SNP detection, genome/transcriptome reconstruction, and metagenomics (Rodrigue et al. 2010), whereas GS-FLX+ is used for analyses of community compositions (see main text). Both methods accommodate the use of “tags,” that is, short sequences allowing the simultaneous analysis of several samples. To give a rough estimate, it is possible to run up to 130 samples on a single run of GS-FLX+, which still yields approximately 10000 sequences per sample. Contrarily to GS-FLX+, Illumina does not allow to easily select a region of interest in the genome, which may explain why its usefulness in the assessment of broad ecological patterns is more dubious, although ways to circumvent this limitations are being implemented (Degnan and Ochman 2012). However, this method has been successfully used in the reconstruction of metagenomes, such as the human gut microbiota (Vacharaksa and Finlay 2010), which allows for a broad description of the biodiversity at a local site. However, more targeted studies, that is, ones interested in a given functional gene, or seeking to assess biodiversity through the use of a neutral marker such as ribosomal DNA, would probably be more adequately conducted through GS-FLX+, which is indeed more used in ecology (Fig. 1 of main text).

Finer taxonomic resolution and a better differentiation among organisms is becoming simpler as curated reference data bases are put into place (Huse et al. 2007; Liu et al. 2007), and newer high-throughput technologies are being adapted to enable community surveys (Gilbert 2012). These methods offer more sequence redundancy (each taxon is sequenced more than once), and increased accuracy (sequences have fewer unresolved positions). These features may allow a better resolution, compared with the first widely adapted, and currently most widespread, technology, 454 pyrosequencing (Fig. 1). HTS can also be applied to RNA, to recover the metabolically active part of the community (Leininger et al. 2006). Since 2007, the number of ecological studies making use of HTS and related technologies, especially in the fields of marine biology (Comeau et al. 2011), soil fungi (Opik et al. 2009), and host-associated microbiotas (Vacharaksa and Finlay 2010; Flores et al. 2011) is steadily increasing; see Box 2 for a discussion of some of these examples, which show the various ways in which HTS can be put to the service of ecological and evolutionary questions. All three domains of life can be covered (Brown et al. 2009; Comeau et al. 2011), illustrating the potential of the technique to conduct community studies across broad taxonomical scales. However, as methodological issues will eventually be resolved, the need is now of a conceptual framework for community ecology and biogeography, guiding the use of already existing data, and setting guidelines for the generation of new ones.

Ecologists may now acquire data suitable for investigating mechanisms underlying commonly observed biogeographic and ecological patterns. In this article, we will argue that community ecologists, and not only environmental microbiologists, should further exploit these new molecular tools, as they will help refine our understanding of biogeographic processes. Although such calls were already made in recent years (Poole et al. 2012), and excellently described the technical possibilities offered by these tools (Bik et al. 2012), they rarely went beyond stating the potential usefulness of these methods, which in our opinion hampered their adoption by general (here loosely meaning, neither microbial nor molecular) ecologists. Here, we showcase how HTS can be put in practice by revisiting classical questions pertaining to the distribution and dynamics of ecological diversity. In particular, we start from characterization of *α*-diversity, and scale up to the integration of species interactions in species distribution. Doing so, we highlight how these techniques can rapidly transform modern ecology by bringing new answers general ecologists are concerned about. We also draw attention to how better integration of biogeography and environmental microbiology with classical ecology will help both fields address key issues (see e.g*.,* Box 3).

With more than a decade of technological and bioinformatic developments, all conditions are in place for ecologists and biogeographers to adopt this new methodology, and use it to investigate mechanisms underlying the distribution of diversity at multiple spatial scales. Although these methods are increasingly used in ecology, some current biogeographic questions are left virtually untouched (Fig. 1). For example, we found no record of papers using HTS whose goal was to better characterize the dynamics of a meta-community (i.e., telling apart the importance of local environmental and regional processes as drivers of variations in species abundances across sites). One might ponder the reasons for this apparent lack of interest by general ecologists. In our opinion, it is because HTS has not been explicitly presented with a perspective that would appeal to general ecologists who, unlike microbial or molecular ecologists, do not already appreciate the small and invisible (Johnson et al. 2009). Community ecologists and biogeographers should take notice of this opportunity to engage in the study of key ecological issues through a molecular approach. Here, we will make this point by highlighting which areas of research could receive major contributions using these new molecular tools to their full potential, by paying special attention to how microbial systems, with their advantages and pitfalls, should become part of general ecological thinking. We conclude the paper by highlighting possible ways HTS could push community ecology forward and how cross-disciplinary studies will overcome current conceptual limitations.

## Possible breakthroughs

In a seminal paper, Pedrós-Alió (2006) pointed out that the “everything is everywhere” concept was based on the observation that some cultivable organisms that grow in selective media, in any laboratory, can be isolated anywhere in the world. However, the advent of molecular methods, which detected much more diversity than seen in cultivated strains, gave rise to “the great plate count anomaly,'' and acknowledgment that much fewer than 1% of bacteria, for example, were able to be cultivated. With improved tools in hands, our ability to detect these elusive species continues to increase (Cardenas and Tiedje 2008). The research effort to test biogeographic hypotheses using molecular analysis of microbial community will also increase our knowledge of microbial diversity and its distribution. Notably, are microbes' distribution regulated by the same drivers than macrobes? This would require an assessment of the relative strengths of dispersal limitations, neutral dynamics, and local selection across different systems (Green and Bohannan 2006), which will only emerge through a common effort by microbial ecologists and biogeographers. New data gathered to address this question will help refining theoretical predictions, and may suggest new mechanisms and hypotheses to test (Parnell et al. 2009). The ability to generate large numbers of sequences indeed resulted in the ability to detect organisms with extremely low abundances, and it is no surprise that an early application of next-generation sequencing in ecology was the exploration of the rare biosphere in marine microbes (Sogin et al. 2006). However, a more accurate picture of biodiversity allows one to go well beyond the description of patterns of *α*-diversity. HTS offers more than a simple table of species presence/absence or relative abundances over several sites. In this section, we show how we can now scale up from the description of local diversity to the drivers of species distribution.

**Box 2. Case studies of innovative HTS use in ecology**

HTS have been used to uncover extremely interesting results in community ecology. In this box, we briefly review some of these studies, mostly to illustrate the diversity of questions that can be addressed with this tool. Brown et al. (2009) covered the three domains of life, allowing future work on eukaryotic microbes (Bik et al. 2012). This is an important step, as it marks the end of the partitioning between the ecology of bacteria and eukaryotes, including fungi. The ability to assess all of this diversity at once will result in a better integration of the approaches developed independently on each class of organisms. Opik et al. (2009) used 454 pyrosequencing to assess the ecological specificity of arbuscular mycorrhizal fungi (AMF) in a natural environment. Precise description of this specificity proved to be an elusive object before the use of HTS methods. Their results helped refine the idea that specificity was better defined at the scale of traits rather than species, which greatly changed the way AMF systems are looked at. More recently, Paterson et al. (2010) investigated the genomic signal of co-evolution through whole-genome sequencing using 454 pyrosequencing. They showed that co-evolution resulted in accelerated molecular evolution, which is a major step forward in linking co-evolutionary theory to genomics. HTS have also been used to investigate biogeographic patterns. Koopman and Carstens (2011) sequenced the inquiline community of the carnivorous pitcher plant *Sarracenia alata*, and showed that its phyllogeographical structure closely mimicked the one of the host plant. Finally, Bryant et al. (2012) used pyrosequencing to assess environmental filtering along an environmental gradient, and provided evidence that it acted differently on functional and phylogenetic diversity. All taken together, these studies indicate that innovative studies using HTS are possible. Each of them can be viewed as an important breakthrough in its field, and highlight the potential for high-impact research that lies in a better integration of HTS methods in an ecologist's toolbox.

**Box 3. Example of research questions using HTS**

1.*Phylogenetic conservatism under climate change*. HTS can be used in rapidly changing or deteriorating environments, to assess whether the resilience of species to environmental change is affected by phylogenetic conservatism of functional traits. Through the sequencing of neutral and non-neutral markers, one can follow how the conservatism changes through ecological selection. This will build upon previous results showing functional and taxonomical changes in community structure following abrupt environmental perturbations (Comeau et al. 2011), by explaining how these changes are contingent upon the phylogenetic structure of traits. We expect that communities with a higher trait conservatism (phylogenetic inertia) will have their distributions more strongly affected by changes, unless they have high dispersal abilities.

2. *Co-occurence, abundance co-variance, and species interactions*. Several recent contributions point to the idea that species co-occurence can indicate the existence of a biotic interaction (Araújo et al. 2011b; Gravel et al. 2011). These data are difficult to obtain in nature. Coupled with prior knowledge about, for example*,* feeding relationships between classes of organisms, the ability of HTS to provide site-species abundances matrices can be used to test this framework with a large amount of data (Barberán et al. 2011). This will contribute to the important goal of linking the β-diversity of species and their interactions (Poisot et al. 2012). We expect that co-distribution and co-variation in abundances will be stronger for interacting species, which can potentially lead to a new way of inferring species interactions.

3. *Signature of antagonistic co-evolution in the wild*. Antagonistic co-evolution is extremely difficult to detect in the wild, as it requires (i) a replicated spatial design, (ii) knowledge of traits values, and (iii) measures of the species' impact on one another fitness (Gomulkiewicz et al. 2007). However, Paterson et al. (2010) demonstrated that co-evolution left genomic signatures in key genes of interacting organisms. Through the sequencing of key genes in different locations, or along environmental gradients, HTS can be instrumental in testing the Geographic Mosaic of Coevolution hypothesis (Thompson 2005). In keeping with this hypothesis, we expect to detect stronger signatures of selection in high-productivity (e.g., warmer) environments.

## Facets of biodiversity

Biodiversity can be defined by taxonomic, functional, and phylogenetic components or “facets” (Reiss et al. 2009), all of which are equally important. Unless functional redundancy, which is thought to be the exception in nature (Loreau 2004), is the rule among microbes, accurate quantification of these components is crucial to gain predictive accuracy of ecosystem functioning (Díaz et al. 2007) and response to climate change (Devictor et al. 2010b; Meynard et al. 2011). For large-bodied organisms, these can prove hard to measure simultaneously as they require the integration of different and often heterogeneously coded information. Once presence/absence or abundance of a set of species are known, phylogenetic relationships can be assessed either by gathering data from public sequences databases. Repositories, such as GenBank, DDBJ, or EMBL (Benson et al. 2010), could be used to construct phylogenies, or alternatively supertrees could be build from published phylogenies. Finally, inferring functional diversity often involves relying on databases of functional traits, that is, by querying the average value of traits based on the taxonomical information at hand. These databases may, in addition, be more or less well documented, and more or less accurate. For examples, traits values documented from one location may be different from actual traits values at another location. Although these approaches provide highly valuable insights about the distribution and drivers of diversity, their integration requires much effort to gather the data. It is also worth mentioning that this approach relies on species as the smallest unit, hence overlooking potentially important intra-specific variability (Bolnick et al. 2011; Albert et al. 2012), which the high number of sequences generated by HTS allows approaching through analysis of sequences within taxonomic groups.

**Figure 2:**
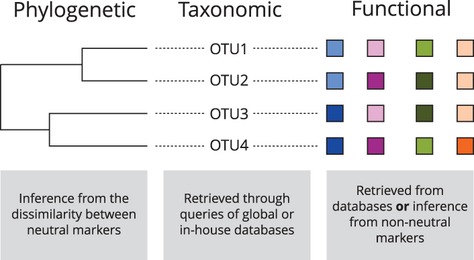
How HTS can give access to the three facets of biodiversity at once. Sequences can be compared to reference databases to obtain taxonomic information. Neutral (here meaning, non-selected) markers (Yang and Rannala 2012) can be used to infer phylogenetic relationships. Finally, either through comparison with databases, or through the sequencing of functional genes, informations about the functional roles of organisms can be gained.

On the other hand, microbial systems analyzed through high-throughput sequencing can make a major contribution as the three facets will become available at once (Fig. 2). As it is already possible to obtain phylogenetic information based on the resulting sequences (see below), the data set in itself already contains both taxonomic and phylogenetic diversity. Moreover, when coupled with basic knowledge of the major taxonomic groups, it is possible to add information about the functions the organisms perform (Dowd et al. 2008). Another way to obtain targeted functional information is to work on a functional gene rather than, or preferably as a complement to, neutral markers (Gilbert et al. 2010; Sun et al. 2011). This begs the question of whether functional traits or functional genes are the relevant unit upon which to base a definition of functional diversity, or at the very least requires rigorous assessment of the association between functional genes and the trait value they confer (Green et al. 2008). A solution to this problem might be to focus on markers providing a high enough phenotypic diversity (Andersen and Lübberstedt 2003). Although what constitutes “traits'' can be defined very broadly according to what is observable of the organisms studied (e.g*.,* Violle et al. 2007), this method allows explicitly grounding it in genetics. Focusing on a hypothesis-based selection of markers can bring information on how organisms respond to environmental change over evolutionary time scales (Feddermann et al. 2010), in addition to the increased predictive power coming with knowledge of functional diversity (Zhang et al. 2012). Ultimately, the development of HTS on non-neutral markers, and the confrontation of neutral versus non-neutral diversity will enable quantification of the structuring impact of niche versus neutral processes (Gravel et al. 2006). One such way to approach this problem would be to compare the distance decay, or temporal autocorrelation, of neutral versus non-neutral diversities (Nekola and White 1999; Morlon et al. 2008; Wetzel et al. 2012). Comparison of this signal between neutral and non-neutral markers will be informative as to the relative importance of neutral versus niche-based processes in the community studied: for example, if similarity between neutral markers decreases faster with distance than similarity of the non-neutral marker, this is indicative of local selection on functional traits.

HTS methods offer interesting access to intra-specific variability by sequencing numerous individuals belonging to the same OTU/species, and expanding the current practices to sequence more than one gene per study. Markers such as rRNA genes, which are commonly used, may not display enough intra-specific variance to do this, but the ever-decreasing costs of HTS will allow increasing the number of markers. High intra-taxon variability is a constant feature of microbial populations, and one that could be easily related to recent conceptual advances in evolutionary ecology linking inter-individual variation to community processes. Recent research emphasized the importance of intra-specific variability for community function (Bolnick et al. 2003), dynamics (Bolnick et al. 2011), and resilience to environmental change (Bolnick and Fitzpatrick 2007). The diversity of intra-specific strategies can buffer the impact of environmental changes (Kremp et al. 2012). Accurately quantifying intra-taxon variability will allow testing recent hypotheses about how species and community structure arise from the accumulation of individuals displaying different specialization and niche overlap (Devictor et al. 2010a; Araújo et al. 2011a; Schreiber et al. 2011). It, however, requires the capacity to assess variability at a large community scale, and HTS appears as an appropriate tool for this.

### Spatio-temporal variability in community structure

Partitioning methods are necessary to understand how diversity, be it taxonomic, functional, or phylogenetic, varies across scales (Tuomisto 2011). The most classical partition is among α, β, and γ components, and there is an active debate about how to best characterize the processes regulating the relationships between them, as it gives direct clues about the community assembly process (Münkemüller et al. 2012). All three facets of biodiversity can be partitioned, and simultaneously described using HTS (Fig. 2). This will become a major advantage for underlying community assembly rules by combining taxonomic, functional, and phylogenetic diversity indices to disentangle different perspectives of metacommunity dynamics from species distribution (Münkemüller et al. 2012).

Additionally, extremely rare species can be detected, which has the potential of opening new fields of research. The definition of what constitutes a rare species varies from study to study, and from system to system. Percentages of 0.01% or 0.1% of the total number of sequences are proposed (Pedrós-Alió 2006; Galand et al. 2009) and the most common 50 species (Comeau et al. 2011), or species representing more than 1% of all sequences (Pedrós-Alió 2006; Galand et al. 2009), were considered abundant. These arbitrary thresholds are sensitive to the total sequence count, so perhaps abundance ranks of OTUs would be more universally applicable. Having a reliable criterion for the limit between abundance and rarity, or adoption a more continuous view of abundance, would allow linking the species abundance to its contribution to, for example, β-diversity (Novotny and Basset 2005; Fontana et al. 2008).

HTS applied to DNA and RNA can be used to separate total community from active communities. Targeting mRNA gives direct access to the putative functions (Xie et al. 2012). This is an unprecedented opportunity to refine predictions of β-diversity patterns. Many microbes are able to form spore and cysts or even remain dormant when growing conditions are poor or environmental conditions adverse. These inactive cells constitute biodiversity store that enables both widespread dispersion and a source of organisms to take advantage of changing environmental conditions (Harding et al. 2011). We would expect that total (DNA, i.e., active, inactive, but also dead cells) community would be more similar across sites than the active (RNA) fraction. This would prove important to integrate predictions of the biodiversity insurance hypothesis in biogeography (Loreau et al. 2003): spatial variation in the dormant species can be integrated to models predicting the changes in ecosystem functions under changing conditions.

### Biotic and abiotic drivers of community structure

#### Species sorting and models of distribution

Modeling species response to global change is among the hottest topics in biogeography at the moment (Richardson 2012). Traditional modeling tools for community ecologists have been ordination techniques such as canonical correspondence analysis and redundancy analysis (Legendre and Legendre 1998). These tools are useful to document species co-distribution, spatial autocorrelation, and test alternative hypotheses of species distribution such as species sorting and dispersal limitations (Gilbert and Lechowicz 2004; Cottenie 2005; Gravel et al. 2008). There has been a shift, however, over the last decade, toward the so-called niche models, or species distribution models. These models aim to elucidate the fundamental relationship between a species range and its environment (Guisan and Thuiller 2005) and they are used to forecast future ranges under various global change scenarios (e.g., Pereira et al. 2010). Despite being heavily criticized for their assumptions such as equilibrium species distribution and no incidence of biotic interactions, they are still useful to provide approximate predictions for natural resource managers. Recent promising developments relaxed some of these assumptions (Kissling et al. 2012; Boulangeat et al. 2012), by accounting explicitely for biotic interactions in the current distribution and co-distribution of species.

Calibrating such models requires accurate data about how species are distributed through space and consequently have not been put to use in microbiology as extensively as they are for vertebrates and plants. Range maps of microbes are difficult to generate because of limited sampling over global scales. Low cost HTS and international coordinated sampling strategies such as carried out for the International Census of Marine Microbes (ICOMM), in addition to the data-mining of other large HTS databases (e.g., RAST, CAMERA and NCBI SRA), will undoubtedly provide insights about microbes biogeography over the next few years. Integrating modeling techniques with the microbiologist toolbox will be extremely useful for predicting vulnerability of microbial communities to global changes such as climate warming, in addition to enable more mechanistic understanding of the drivers of microbial diversity. Perhaps because they were more easily sampled, plants and animals were (and are still) used to derive the core of the theory for community ecology (Scheiner and Willig 2011). Microbes, despite their widespread distribution, abundance, and importance for functioning, were neglected. As a consequence, the core of community ecology theory is disconnected from microbial systems. As such, (1) it is not clear which classical results of community ecology holds for microbes and, (2) the investigations of this is hampered by the fact that sampling of microbial populations was not always framed in the context of ecological questions.

#### Biotic interactions and networks of co-occurrence

The current framework for species distribution models, whether using correlative or process-based approaches, relies largely on abiotic drivers. There is on-going work to add biotic drivers and population dynamics to predict species range (Kissling et al. 2012), but there is currently no good model, nor unifying theory, to scale up individual species predictions to the community level (Lurgi et al. 2012). Adding species interactions to species distribution models and biodiversity scenarios is by no means trivial, as most ecological systems are often quite complex. There are nonetheless promising avenues derived from the study of co-occurrence patterns. It has long been hypothesized that if two species co-occur less frequently than expected by chance alone, they must interact negatively or have in the past (Cody and Diamond 1975; Gotelli and Graves 1996). More recently, Araújo et al. (2011b) developed species co-occurrence networks based on the hypothesis that if two species are found more often together than by chance alone, they are also more likely to interact. The increased ability to define finer taxonomic groups using HTS compared with traditional methods will refine our knowledge of the co-occurrence patterns, thus testing the usefulness of theoretical predictions. Note also that using genetic tools to approach the problem of species co-occurrence provides a major advance for understanding of co-evolution. Thompson (2005) postulated the existence of geographic mosaics of reciprocal selection, which are notoriously difficult to detect (Gomulkiewicz et al. 2007). Paterson et al. (2010) used HTS to detect genomic signature of reciprocal selection in a bacteria–phage system, by comparing site-specific mutation rates of viruses and bacteria in evolved versus co-evolved treatments. Looking for these clues of co-evolutionary dynamics in natural environments would allow testing this framework in an unprecedented way, and pave the way to an integrated theory of evolutionary biogeography (Urban et al. 2008; Leibold et al. 2010).

Co-occurrence patterns were recently used to improve species distribution models and to reveal the fundamental niche from realized distributions (Boulangeat et al. 2012). It is almost impossible to observe in situ interactions among microbes and consequently, we have to rely on indirect methods such as these to evaluate them. The high resolution of HTS now makes this type of analysis possible (Beman et al. 2011), which will open new possibilities to our understanding of microbe distribution and community ecology. Moreover, the study of microbes' co-distribution will be innovative for ecologists because of their inherent characteristics, such as high turnover rate, dispersal, and evolutionary responses. We still have no clear idea of what co-distribution we should expect and their study should open new perspectives in biogeography.

## Overcoming the methodological issues

### Computational and conceptual issues

Different information can be obtained from the HTS data. Sequences can be used directly or clustered as Operational Taxonomic Units (OTU), and taxonomic information can be inferred on both of these levels of organization. The existence of these two possibilities begs the question of the appropriate level at which diversity should be described and analyzed in such data sets. Only rarely sequences are directly used in HTS data analysis, partly because of the danger that sequencing errors could inflate biodiversity estimates (Acinas et al. 2004), On the other hand, using OTUs (1) could result in losing some information such as intra-specific variability, and (2) can miscategorize a sequence to an OTU during the clustering stage, depending on the clustering algorithm used (Schloss et al. 2009). The use of sequences or OTUs may lead to different insights and will be influenced by the hypothesis being tested. Even in the absence of a consensus on the right scale of observation, common sense indicates that studies involving genetic differentiation between populations, such as studies of local adaptation, should stay focused on sequences. It is, however, important to conduct a screening of sequences to remove chimeras or other artifacts (most HTS software provides ways to check for this). This approach accounts for both intra- and inter-group variability, which are necessary to account for in such studies (Kawecki and Ebert 2004). OTUs can be used either when information carried by intra-taxon variability can safely be overlooked, such as studies of species sorting over an environmental gradient, or when the confidence in taxonomic attribution is low, in which case one might choose to avoid the risk of wrong identification of the species or genus.

Regardless of the level of data aggregation chosen, HTS data can be summarized in a community matrix (a site-by-taxon presence/absence or abundance table), which can be analyzed through null models (Gotelli 2000). These allow understanding which features of the communities represent statistically significant departures from random expectations. Null models help revealing significant structure in species distribution, even in the absence of strong theoretical predictions, by comparing to the expected distribution from chance acting alone. Although this approach can be deemed inferential, applying such methodology to HTS data will enhance ecologists' understanding of microbial distribution. In communities with an important turnover, for example, it might be tempting to determine if the variations in the taxa pool are lower (indicating environmental filtering) or not (indicating stochasticity) than expected by random variability. It should be noted that instead of using taxa, the community matrix can be constructed with functions, which would allow separating the importance of the taxonomic versus functional composition of the community.

Still, a major methodological uncertainty in measuring microbial diversity is the quantification of evenness, that is, switching from the presence/absence to abundance data. Gihring et al. (2012) reinforced the idea that evenness measures like Simpson's or Shannon's indices cannot be applied to data sets with unequal species counts between tags (essentially, the sequencing process yields a different number of sequences across samples), and recommend that sequences be randomly removed to obtain an equal number of sequences per data set. Such measures have been corrected for unequal richness long ago (Routledge 1983); simply put, it is possible to calculate the maximal expected value given the number of species, and the resulting evenness is expressed as a fraction of this maxima. Even if it were not the case, one can apply a permutative approach, and repeat the random draw of sequences a large number of times. If anything, the existence of this debate reinforces the mutual benefits that would be derived from an increased dialog across disciplines.

There is, however, a more pressing issue, namely the usability of these measures based on HTS abundance data. Implicitly, quantification of evenness makes the assumption that the “count” for each species/OTU is a proportional and unbiased proxy to abundance. Quantification through HTS was shown to be highly sensitive to biases in a dilution experiment (Amend et al. 2010). The authors assembled a community of known abundances, diluted it, and estimated the abundances in the diluted samples through 454 pyrosequencing. Their analysis revealed that increasingly diluted samples yielded different community structures, casting doubt on the quantitative aspects of the sequencing method. It should be noted that all *R*^2^ for the ability to quantify species abundances known from the original community fell within the 0.54–0.96 range, which are still relatively high values. In addition, not all species have the same number of genomic copies of the marker gene (Chaffron et al. 2010), or different primer affinities (Lovejoy and Potvin 2010). This leads to some OTUs being over-represented in the original sample, a fact susceptible to be amplified through PCR. In bacteria, heterogeneity in gene copy number is well described as a covariate of ecological strategy (Klappenbach et al. 2000; Stevenson and Schmidt 2004), which can introduce extremely strong biases in the association between taxonomic and functional biodiversity. Although it may seem extremely conservative, we suggest that until these biases are corrected, accounted for, or understood, ecologists be careful in their use of quantitative data, failing what there is a risk to estimate α or β diversity on the basis of biased data. To some extent, this problem could be circumvented using a method like bootstrap through intra-OTU resampling, but the computational difficulty of doing so probably makes it an un-attainable goal for current software, if one is to generate enough draws to get a satisfactory statistical power.

### HTS-based community phylogenetics

Next-generation sequencing is most often conducted with markers having a long history of being used in phylogenetic analyses, typically hyper-variable regions of SSU rRNA genes. Phylogenetic information offers more than just increasing the taxonomic resolution of microbial community surveys; it provides an opportunity for ecologists to better estimate the forces that shape these communities, and to more accurately quantify their relative impacts (Chamberlain et al. 2012). However, although use of phylogeny-based measures such as the Phylogenetic Dissimilarity (Faith 1992) is increasing, most HTS-based studies of microbial assemblages, so far, do not directly investigate these forces, and stay largely focused on community α and β diversities measured on taxonomic information. Using only the presence/absence or relative abundance patterns and associated taxonomic distributions is unfortunate, as such approach under-exploits the information enclosed in these large sequence data sets. Moreover, inferring ecological processes is difficult because of the lack of direct relatedness metrics between co-occurring OTUs based on mapping taxonomic predictions.

A powerful approach to directly access processes structuring microbial communities lies in the reconciliation of evolutionary biology and ecology. Community phylogenetic analysis, that is, the use of phylogenetic information about the relatedness of co-occurring OTUs to determine properties of community structure, was proposed a decade ago and gained in prominence since (Webb et al. 2002, 2006; Cadotte et al. 2010). This approach is useful as it allows disentangling the impact of traits and evolutionary history on community structure, in a context where not all traits display phylogenetic conservatism. Cavender-Bares et al. (2009), for example, emphasize that different phylogenetic structure of traits (indicating, e.g., brownian evolution, convergence, or strong conservatism), resulted in different associations among the phylogenetic, functional, and taxonomic structure of the community. This potential discrepancy led to a rapid development of methodologies (see Mouquet et al. 2012; for a review), culminating with the availability of measures of community structure, and dissimilarity grounded in phylogenetic information. The latest generation of these methods partitions taxonomic and phylogenetic components at all spatial scales (Ives and Helmus 2010; Morlon et al. 2011). Despite this, they are not yet widely applied in HTS-based ecological studies. Ecophylogenetics have not percolated the field of HTS-based ecology due to perceived methodological and theoretical issues. These include the computational requirements needed to reconstruct phylogenetic trees from large-scale HTS data sets using likelihood or Bayesian inference methods, and the misconception that short HTS sequences lack sufficient phylogenetic signal for tree reconstruction and inferences of ecological processes. These issues and concerns no longer stand; very large phylogenetic trees are now routinely reconstructed, thanks to novel implementations of probabilistic tree reconstruction methods, such as FastTree, PhyloBayes, or specific modes of RAxML. These softwares provide fast yet robust phylogenetic tree inference over thousands of possibly short sequences. Moreover, several studies have shown that hyper-variable regions of the SSU rRNA gene (arguably the most widespread marker in HTS and non-HTS studies alike) sequence possess enough phylogenetic signal to reflect niche adaptation, and that such sequences can be used to infer ecological processes at play in structuring communities (Acinas et al. 2004; Johnson et al. 2006; Koopman and Carstens 2011). Future efforts to determine which sets of other markers are also suitable will increase the usability of these methods in HTS studies.

Next generation of HTS-based ecological studies with a phylogenetic perspective can also benefit from an important research avenue – the investigation of the role of past stochastic versus deterministic processes in structuring communities. Random processes, such as dispersal, can now be evaluated based on null hypotheses such as testing phylogenetic structure of a given community against the structure of a randomized phylogeny. These recent developments in constrained randomization procedures of phylogenies, coupled to the statistical testing of null models, furthered our understanding of the role of stochastic processes in shaping communities (Kembel 2009). The usefulness of these methods will increase with the number of sequences they can accommodate. Applying them to HTS data will be instrumental in developing better insights about the processes shaping diversity. We foresee that with the increase in sequence length and quality, and decreases of the costs, HTS data will boost the field of community phylogenetics forward importantly in the coming years. Finally, it is possible to go full-circle on these questions, by laying out explicit hypotheses about the role of phylogenetic conservatism on current species distributions. Diniz-Filho and Bini (2008) show that the importance of conservatism in habitat selection traits, when coupled with prior knowledge of dispersal ability, is a predictor of community responses and re-assembly under climate change. Because microbes (1) evolve faster than most other organism, (2) are present in extremely steep environmental gradients, or rapidly deteriorating environments, and (3) are well studied using HTS methods, they offer the opportunity to develop meaningful collaborations between microbial ecologists and general ecologists on these topics.

### Data sharing and indexing

Novel approaches to the analysis of HTS data will require the ability to integrate information from different data sets (notably when reconstructing species ranges). This in turn requires two things: (1) an integrated database or network of repositories for HTS data (Sun et al. 2011), and (2) cautious definition of metadata. These conditions must be met in order to access not only a sequence, but information about its environment (e.g*.,* the MIENS specification). Such a specification should also cover which genes, and which portions of the genes to use as markers, enabling comparison among studies. A minima, records about geographic position, time of sampling, and a small set of environmental data (e.g*.,* depth, salinity, and temperature for marine environments, or pH and type of vegetation coverage for soils) should be associated with each record. It is highly probable that if this basic information was added to sequences deposited in the CAMERA database or a similar initiative, interesting biogeographic patterns could be investigated. Having rigorous metadata associated with each sequences will offer the tremendous opportunity to link these and other databases (Deans et al. 2012; Parr et al. 2012). It will allow extensive data-mining projects, and will leverage the important amount of existing data. Entirely, new research avenues will open up. Ecologists routinely collect such metadata during their investigations, and as such will be likely to contribute relevant environmental information to these databases, extending their usefulness for all users. While HTS is undoubtedly an extremely potent tool to analyze local community structure, coupling it with exhaustive metadata in an easy to access database will allow much more creative approaches. It will ultimately become realistic to reconstruct the geographic distribution of a species, and to look for variations in environmental traits explaining its presence or absence. Recent developments in extensive and automated database querying using free software will decrease the quantity of effort needed to integrate across these sources of information (e.g., the ROpenSci project). Such information is essential to get a clear understanding of drivers of microbial biogeography and to eventually add microbes to biodiversity scenarios (Gormley et al. 2011). Despite their importance for ecosystem functioning and clear evidence of the existence of a strong, environment driven biogeographic signal, both in soils and oceans, microbes are systematically ignored in such modeling studies (Pereira et al. 2010). This perhaps come out of neglect from ecologists, or because of the still standing conception that they are distributed everywhere.

## Conclusions

Biogeography predicts the consequences of global changes on earths' environments through a deeper understanding of the mechanisms structuring the spatial distribution of diversity across scales of organization (phylogenetic, taxonomic, and functional). Some of the most exciting questions of this field require a large amount of data, which can be expensive and difficult to generate with large-bodied organisms. By using HTS, ecologists will be able to generate such data in a cost-efficient and rapid way for microbes. These organisms helped us (in a laboratory setting) understanding the underlying mechanisms of ecology and evolution (Buckling et al. 2009; Weitz et al. 2013). The same can be said of them from natural environments, provided that we have access to a good enough way to describe their diversity. It is our intuition that some questions can only be addressed at a large scale by relying on next-generation methods. It could help, for instance, to understand species range shift by separating effects of local adaptation, tolerance, dispersal, and rate of adaptation to novel environments (Leibold et al. 2010).

A biogeographic survey, such as undertook by Comeau et al. (2011), can help us understand how communities respond to large-scale events (in this case, the record sea ice minimum in the Arctic Ocean), by analyzing DNA from independent studies, carried out in the same biogeographic region over time. This study surely illustrates the potential of integrating data sets from several samplings to paint a broader picture of changing ecosystems. More recently, Yu et al. (2012) showed how the integration of traditional and HTS methods made for a rapid way to assess arthropod biodiversity, both taxonomic and phylogenetic. The ability to deploy high precision methods in a short amount of time will become instrumental to react rapidly to environmental emergencies, some of which made the news over the last 2 years (Campagna et al. 2011; Ihaksi et al. 2011). In the case of the Deepwater Horizon oil spill, resident petroleum degrading bacteria were accounted for in the strategies implemented to deal with the crisis, stressing why a good understanding of the taxonomic and functional composition of the community can be crucial. With the decrease in costs, the increase in the number of facilities equipped with HTS facilities, and the availability of software to rapidly analyze the data, we see an opportunity for conservationists to rely more heavily on these tools in the future.

After reviewing the different situations in which HTS can help biogeography move forward, it is clear that progresses will come as a result of reinforced collaboration between environmental microbiologists and ecologists. A possible research agenda to achieve this integration can be drafted from the points we discussed here. From the microbiology side, we identify two important steps. First, there is an urgent need to develop a central repository with relevant metadata, so that we could eventually build up range maps and perform species distribution models. Integrating pre-existing data sets in it will already be a significant improvement of the current situation. The emergence of locally maintainable databases (Langille et al. 2012) strikes us as a particularly counter-productive one, unless these databases are conceived around the idea of facilitation programmatic access. Splitting the data between research groups and institutions will hamper our ability to build upon the important quantity of information already gathered. This requires efforts in terms of maintenance, and the development of API and portals to integrate across heterogeneous databases. Second, data should be analyzed with a hypothesis-based approach. This will be greatly helped by ecologists being more vocal and engaging about what are the major questions in biogeography, so that they can be better integrated into the work flow of microbial ecologists.

In addition, there should be an increased effort to develop an overarching theory that will link the spatial distribution of diversity from genes to functions, (Whitham et al. 2006; Burke et al. 2011; Miner et al. 2012). These steps may seem large ones at first, but most of the groundwork is already done, and the focus should now switch to integration between concepts and methodologies. Finally, HTS will gain in popularity through joint efforts by all scientists involved in its use, particularly with regard to computing and training. The development of data analysis procedures, so as to facilitate data analysis for non-specialists, should account for the needs of ecologists. Vast libraries of community ecology methods have been developed for the most popular statistical softwares (see, e.g., Oksanen et al. 2009), and the advanced analyses they allow can easily be integrated to existing HTS analysis software. Similarly, while free, open-source tools already exist to analyze the phylogenetic structure of communities (Kembel et al. 2010), it is likely that they will not nicely scale up to the amount of data generated by HTS. In this regard, the increased availability of massively parallel GPU-based tools, and the relative ease with which this hardware can be programmed, will be of invaluable help (Manavski and Valle 2008). There is, finally, an increased need for training. This needs not only covering the experimental part of HTS but also provides a crash-course in data analysis from an ecologist point of view. In brief, the opportunity for a joint effort is tremendous, and we foresee that it will greatly increase the quality of ecological science produced through HTS, ultimately furthering our understanding of biological diversity.

## References

[b1] Acinas SG, Klepac-Ceraj V, Hunt DE, Pharino C, Ceraj I, Distel DL (2004). Fine-scale phylogenetic architecture of a complex bacterial community. Nature.

[b2] Albert CH, Boulangeat F, de Bello I, Pellet G, Lavorel S, Thuiller W (2012). On the importance of intraspecific variability for the quantification of functional diversity. Oikos.

[b3] Amend AS, Seifert KA, Bruns TD (2010). Quantifying microbial communities with 454 pyrosequencing: does read abundance count?. Mol. Ecol.

[b4] Andersen JR, Lübberstedt T (2003). Functional markers in plants. Trends Plant Sci.

[b5] Araújo MS, Bolnick DI, Layman CA (2011a). The ecological causes of individual specialisation. Ecol. Lett.

[b6] Araújo MB, Rozenfeld A, Rahbek C, Marquet PA (2011b). Using species co-occurrence networks to assess the impacts of climate change. Ecography.

[b7] Barberán A, Bates ST, Casamayor EO, Fierer N (2011). Using network analysis to explore co-occurrence patterns in soil microbial communities. ISME J.

[b8] Beman JM, Steele JA, Fuhrman JA (2011). Co-occurrence patterns for abundant marine archaeal and bacterial lineages in the deep chlorophyll maximum of coastal California. ISME J.

[b9] Benson DA, Karsch-Mizrachi I, Lipman DJ, Ostell J, Sayers EW (2010). GenBank. Nucleic Acids Res.

[b10] Bik HM, Porazinska DL, Creer S, Caporaso JG, Knight R, Thomas WK (2012). Sequencing our way towards understanding global eukaryotic biodiversity. Trends Ecol. Evol.

[b11] Bolnick DI, Fitzpatrick BM (2007). Sympatric speciation: models and empirical evidence. Annu. Rev. Ecol. Evol. Syst.

[b12] Bolnick DI, Svanbäck R, Fordyce JA, Yang LH, Davis JM, Hulsey CD (2003). The ecology of individuals: incidence and implications of individual specialization. Am. Nat.

[b13] Bolnick DI, Amarasekare P, Araújo MS, Bürger R, Levine JM, Novak M (2011). Why intraspecific trait variation matters in community ecology. Trends Ecol. Evol.

[b14] Boulangeat I, Gravel D, Thuiller W (2012). Accounting for dispersal and biotic interactions to disentangle the drivers of species distributions and their abundances. Ecol. Lett.

[b15] Brown MV, Philip GK, Bunge JA, Smith MC, Bissett A, Lauro FM (2009). Microbial community structure in the North Pacific ocean. ISME J.

[b16] Bryant JA, Stewart FJ, Eppley JM, DeLong EF (2012). Microbial community phylogenetic and trait diversity declines with depth in a marine oxygen minimum zone. Ecology.

[b17] Buckling A, MacLean RC, Brockhurst MA, Colegrave N (2009). The Beagle in a bottle. Nature.

[b18] Burke C, Steinberg PD, Rusch D, Kjelleberg S, Thomas T (2011). Bacterial community assembly based on functional genes rather than species. Proc. Nat. Acad. Sci. U.S.A.

[b19] Cadotte MW, Jonathan Davies T, Regetz J, Kembel SW, Cleland E, Oakley TH (2010). Phylogenetic diversity metrics for ecological communities: integrating species richness, abundance and evolutionary history. Ecol. Lett.

[b20] Campagna C, Short FT, Polidoro BA, McManus R, Collette BB, Pilcher NJ (2011). Gulf of Mexico Oil Blowout Increases Risks to Globally Threatened Species. Bioscience.

[b21] Cardenas E, Tiedje JM (2008). New tools for discovering and characterizing microbial diversity. Curr. Opin. Biotechnol.

[b22] Cavender-Bares J, Kozak KH, Fine PVA, Kembel SW (2009). The merging of community ecology and phylogenetic biology. Ecol. Lett.

[b23] Chaffron S, Rehrauer H, Pernthaler J, von Mering C (2010). A global network of coexisting microbes from environmental and whole-genome sequence data. Genome Res.

[b24] Chamberlain SA, Hovick SM, Dibble CJ, Rasmussen NL, Van Allen BG, Maitner BS (2012). Does phylogeny matter? Assessing the impact of phylogenetic information in ecological meta-analysis. Ecol. Lett.

[b25] Cody M, Diamond JM (1975). Ecology and Evolution of communities.

[b26] Comeau AM, Li WKW, Tremblay J-E, Carmack EC, Lovejoy C (2011). Arctic Ocean Microbial Community Structure before and after the 2007 Record Sea Ice Minimum. PLoS ONE.

[b27] Cottenie K (2005). Integrating environmental and spatial processes in ecological community dynamics. Ecol. Lett.

[b28] De Wit R, Bouvier T (2006). ‘Everything is everywhere, but, the environment selects’; what did Baas Becking and Beijerinck really say?. Environ. Microbiol.

[b29] Deans AR, Yoder MJ, Balhoff JP (2012). Time to change how we describe biodiversity. Trends Ecol. Evol.

[b30] Degnan PH, Ochman H (2012). Illumina-based analysis of microbial community diversity. ISME J.

[b31] Devictor V, Clavel J, Julliard R, Lavergne S, Mouillot D, Thuiller W (2010a). Defining and measuring ecological specialization. J. Appl. Ecol.

[b32] Devictor V, Mouillot D, Meynard CN, Jiguet F, Thuiller W, Mouquet N (2010b). Spatial mismatch and congruence between taxonomic, phylogenetic and functional diversity: the need for integrative conservation strategies in a changing world. Ecol. Lett.

[b33] Díaz S, Lavorel S, Quétier F, de Bello F, Grigulis K, Robson TM (2007). Incorporating plant functional diversity effects in ecosystem service assessments. Proc. Nat. Acad. Sci. U.S.A.

[b34] Diniz-Filho JAF, Bini LM (2008). Macroecology, global change and the shadow of forgotten ancestors. Glob. Ecol. Biogeogr.

[b35] Dowd SE, Sun Y, Secor PR, Rhoads DD, Wolcott BM, James GA (2008). Survey of bacterial diversity in chronic wounds using pyrosequencing, DGGE, and full ribosome shotgun sequencing. BMC Microbiol.

[b36] Faith D (1992). Conservation evaluation and phylogenetic diversity. Biol. Conserv.

[b37] Feddermann N, Finlay R, Boller T, Elfstrand M (2010). Functional diversity in arbuscular mycorrhiza – the role ofgene expression, phosphorous nutrition and symbiotic efficiency. Fungal Ecol.

[b38] Flores GE, Bates ST, Knights D, Lauber CL, Stombaugh J, Knight R (2011). Microbial biogeography of public restroom surfaces. PLoS ONE.

[b39] Fontana G, Ugland KI, Gray JS, Willis TJ, Abbiati M (2008). Influence of rare species on beta diversity estimates in marine benthic assemblages. J. Exp. Mar. Biol. Ecol.

[b40] Galand PE, Casamayor EO, Kirchman DL, Lovejoy C (2009). Ecology of the rare microbial biosphere of the Arctic Ocean. Proc. Nat. Acad. Sci. U.S.A.

[b41] Gihring TM, Green SJ, Schadt CW (2012). Massively parallel rRNA gene sequencing exacerbates the potential for biased community diversity comparisons due to variable library sizes. Environ. Microbiol.

[b42] Gilbert B (2012). Joint consequences of dispersal and niche overlap on local diversity and resource use. J. Ecol.

[b43] Gilbert B, Lechowicz MJ (2004). Neutrality, niches, and dispersal in a temperate forest understory. Proc. Nat. Acad. Sci. U.S.A.

[b44] Gilbert JA, Field D, Swift P, Thomas S, Cummings D, Temperton B (2010). The taxonomic and functional diversity of microbes at a temperate coastal site: a ‘multiomic’ study of seasonal and diel temporal variation. PLoS ONE.

[b45] Gomulkiewicz R, Drown DM, Dybdahl MF, Godsoe W, Nuismer SL, Pepin KM (2007). Dos and don'ts of testing the geographic mosaic theory of coevolution. Heredity.

[b46] Gormley AM, Forsyth DM, Griffioen P, Lindeman M, Ramsey DSL, Scroggie MP (2011). Using presence-only and presence-absence data to estimate the current and potential distributions of established invasive species. J. Appl. Ecol.

[b47] Gotelli NJ (2000). Null model analysis of species co-occurrence patterns. Ecology.

[b48] Gotelli NJ, Graves GR (1996). Null models in ecology.

[b49] Gravel D, Canham CD, Beaudet M, Messier C (2006). Reconcilingniche and neutrality: the continuum hypothesis. Ecol. Lett.

[b50] Gravel D, Beaudet M, Messier C (2008). Partitioning the factors of spatial variation in regeneration density of shade-tolerant tree species. Ecology.

[b51] Gravel D, Canard E, Guichard F, Mouquet N (2011). Persistence increases with diversity and connectance in trophic metacommunities. PLoS ONE.

[b52] Green J, Bohannan BJM (2006). Spatial scaling of microbial biodiversity. Trends Ecol. Evol.

[b53] Green JL, Bohannan BJM, Whitaker RJ (2008). Microbial biogeography: from taxonomy to traits. Science.

[b54] Guisan A, Thuiller W (2005). Predicting species distribution: offering more than simple habitat models. Ecol. Lett.

[b55] Harding T, Jungblut AD, Lovejoy C, Vincent WF (2011). Microbesin higharctic snow and implications for the cold biosphere. Appl. Environ. Microbiol.

[b56] Huse SM, Huber JA, Morrison HG, Sogin ML, Welch DM (2007). Accuracy and quality of massively parallel DNA pyrosequencing. Genome Biol.

[b57] Ihaksi T, Kokkonen T, Helle I, Jolma A, Lecklin T, Kuikka S (2011). Combining conservation value, vulnerability, and effectiveness of mitigation actions in spatial conservation decisions: an application to coastal oil spill combating. Environ. Manag.

[b58] Ives AR, Helmus MR (2010). Phylogenetic Metrics of Community Similarity. Am. Nat.

[b59] Johnson Z, Zinser E, Coe A (2006). Niche partitioning among Prochlorococcus ecotypes along ocean-scale environmental gradients. Science.

[b60] Johnson KP, Malenke JR, Clayton DH (2009). Competition promotes the evolution of host generalists in obligate parasites. Proc. Roy. Soc. B Biol. Sci.

[b61] Kawecki TJ, Ebert D (2004). Conceptual issues in local adaptation. Ecol. Lett.

[b62] Kembel SW (2009). Disentangling niche and neutral influences on community assembly: assessing the performance of community phylogenetic structure tests. Ecol. Lett.

[b63] Kembel SW, Cowan PD, Helmus MR, Cornwell WK, Morlon H, Ackerly DD (2010). Picante: R tools for integrating phylogenies and ecology. Bioinformatics.

[b64] Kissling WD, Dormann CF, Groeneveld J, Hickler T, Kühn I, McInerny GJ (2012). Towards novel approaches to modelling biotic interactions in multispecies assemblages at large spatial extents. J. Biogeogr.

[b65] Klappenbach JA, Dunbar JM, Schmidt TM (2000). rRNA operon copy number reflects ecological strategies of bacteria. Appl. Environ. Microbiol.

[b66] Koopman MM, Carstens BC (2011). The microbial phyllogeography of the carnivorous plant sarracenia alata. Microb. Ecol.

[b67] Kremp A, Godhe A, Egardt J, Dupont S, Suikkanen S, Casabianca S (2012). Intraspecific variability in the response of bloom-forming marine microalgae to changed climate conditions. Ecol. Evol.

[b68] Langille MGI, Laird MR, Hsiao WWL, Chiu TA, Eisen JA, Brinkman FSL (2012). MicrobeDB: a locally maintainable database of microbial genomic sequences. Bioinformatics.

[b69] Legendre P, Legendre L (1998). Numerical ecology.

[b70] Leibold MA, Holyoak M, Mouquet N, Amarasekare P, Chase JM, Hoopes MF (2004). The metacommunity concept: a framework for multi-scale community ecology. Ecol. Lett.

[b71] Leibold MA, Economo EP, Peres-Neto P (2010). Metacommunity phylogenetics: separating the roles of environmental filters and historical biogeography. Ecol. Lett.

[b72] Leininger S, Urich T, Schloter M, Schwark L, Qi J, Nicol GW (2006). Archaea predominate among ammonia-oxidizing prokaryotes in soils. Nature.

[b73] Liu Z, Lozupone C, Hamady M, Bushman FD, Knight R (2007). Short pyrosequencing reads suffice for accurate microbial community analysis. Nucleic Acid Res.

[b74] Loreau M (2004). Does functional redundancy exist?. Oikos.

[b75] Loreau M, Mouquet N, Gonzalez A (2003). Biodiversity as spatial insurance in heterogeneous landscapes. Proc. Nat. Acad. Sci. U.S.A.

[b76] Lovejoy C, Potvin M (2010). Microbial eukaryotic distribution in a dynamic Beaufort Sea and the Arctic Ocean. J. Plankton Res.

[b77] Lurgi M, Lopez BC, Montoya JM (2012). Novel communities from climate change. Philos. Trans. R. Soc. B Biol. Sci.

[b78] Manavski SA, Valle G (2008). CUDA compatible GPU cards as efficient hardware accelerators for Smith-Waterman sequence alignment. BMC Bioinformatics.

[b79] Meynard CN, Devictor V, Mouillot D, Thuiller W, Jiguet F, Mouquet N (2011). Beyond taxonomic diversity patterns: how do α, β and γ components of bird functional and phylogenetic diversity respond to environmental gradients across France?. Glob. Ecol. Biogeogr.

[b80] Miner BE, Pfrender L, De Meester ME, Lampert W, Hairston NG (2012). Linking genes to communities and ecosystems: Daphnia as an ecogenomic model. Proc. Roy. Soc. B Biol. Sci.

[b81] Morlon H, Chuyong G, Condit R, Hubbell S, Kenfack D, Thomas D (2008). A general framework for the distance-decay of similarity in ecological communities. Ecol. Lett.

[b82] Morlon H, Schwilk DW, Bryant JA, Marquet PA, Rebelo AG, Tauss C (2011). Spatial patterns of phylogenetic diversity. Ecol. Lett.

[b83] Mouquet N, Devictor V, Meynard CN, Munoz F, Bersier L-F, Chave J (2012). Ecophylogenetics: advances and perspectives. Biol. Rev. Camb. Philos. Soc.

[b84] Münkemüller T, Meynard F, de Bello CN, Gravel D, Lavergne S, Mouillot D (2012). From diversity indices to community assembly processes: a test with simulated data. Ecography.

[b85] Myllykangas S, Buenrostro J, Ji HP, Rodríguez-Ezpeleta N, Hackenberg M, Aransay AM (2012). Overview of Sequencing Technology Platforms. Bioinformatics for High Throughput Sequencing.

[b86] Nekola JC, White PS (1999). The distance decay of similarity in biogeography and ecology. J. Biogeogr.

[b87] Novotny V, Basset Y (2005). Host specificity of insect herbivores in tropical forests. Proc. Roy. Soc. B Biol. Sci.

[b88] O'Dwyer JP, Green JL (2010). Field theory for biogeography: a spatially explicit model for predicting patterns of biodiversity. Ecol. Lett.

[b89] Oksanen J, Kindt R, Legendre P, O'Hara B, Simpson GL, Solymos P (2009). vegan: Community Ecology Package.

[b90] Opik M, Metsis M, Daniell TJ, Zobel M, Moora M (2009). Large-scale parallel 454 sequencing reveals host ecological group specificity of arbuscular mycorrhizal fungi in a boreonemoral forest. New Phytol.

[b91] Parnell JJ, Crowl TA, Weimer BC, Pfrender ME (2009). Biodiversity in microbial communities: system scale patterns and mechanisms. Mol. Ecol.

[b92] Parr CS, Guralnick R, Cellinese N, Page RDM (2012). Evolutionary informatics: unifying knowledge about the diversity of life. Trends Ecol. Evol.

[b93] Paterson S, Vogwill T, Buckling A, Benmayor R, Spiers AJ, Thomson NR (2010). Antagonistic coevolution accelerates molecular evolution. Nature.

[b94] Pedrós-Alió C (2006). Marine microbial diversity: can it be determined?. Trends Microbiol.

[b95] Pereira HM, Leadley PW, Proença V, Alkemade R, Scharlemann JPW, Fernandez-Manjarrés JF (2010). Scenarios for global biodiversity in the 21st century. Science.

[b96] Poisot T, Canard E, Mouillot D, Mouquet N, Gravel D (2012). The dissimilarity of species interaction networks. Ecol. Lett.

[b97] Poole AM, Stouffer DB, Tylianakis JM (2012). ‘Ecosystomics’: ecology by sequencer. Trends Ecol. Evol.

[b98] Purdy KJ, Hurd PJ, Moya-Laraño J, Trimmer M, Oakley BB, Woodward G (2010). Systems biology for ecology. in Advances in Ecological Research.

[b99] Reiss J, Bridle JR, Montoya JM, Woodward G (2009). Emerging horizons in biodiversity and ecosystem functioning research. Trends Ecol. Evol.

[b100] Richardson DM (2012). Conservation biogeography: what's hot and what's not?. Divers. Distrib.

[b101] Rodrigue S, Materna AC, Timberlake SC, Blackburn MC, Malmstrom RR, Alm EJ (2010). Unlocking short read sequencing for metagenomics. PLoS ONE.

[b102] Routledge RD (1983). Evenness indices: are any admissible? En. Oikos.

[b103] Scheiner SM, Willig MR (2011). The Theory of Ecology.

[b104] Schloss PD, Westcott SL, Ryabin T, Hall JR, Hartmann M, Hollister EB (2009). Introducing mothur: open-source, platform-independent, community-supported software for describing and comparing microbial communities. Appl. Environ. Microbiol.

[b105] Schreiber SJ, Bürger R, Bolnick DI (2011). The community effects of phenotypic and genetic variation within a predator population. Ecology.

[b106] Sogin ML, Morrison HG, Huber JA, Welch DM, Huse SM, Neal PR (2006). Microbial diversity in the deep sea and the underexplored “rare biosphere”. Proc. Nat. Acad. Sci.

[b107] Stevenson BS, Schmidt TM (2004). Life history implications of rRNA gene copy number in Escherichia coli life history implications of rRNA gene copy number in Escherichia coli. Appl. Environ. Microbiol.

[b108] Sun Y, Wolcott RD, Dowd SE, Kwon YM, Ricke SC (2011). Tag-Encoded FLX Amplicon Pyrosequencing for the Elucidation of Microbial and Functional Gene Diversity in Any Environment. High-Throughput Next Generation Sequencing. Methods in Molecular Biology.

[b109] Tedersoo L, Nilsson RH, Abarenkov K, Jairus T, Sadam A, Saar I (2010). 454 Pyrosequencing and Sanger sequencing of tropical mycorrhizal fungi provide similar results but reveal substantial methodological biases. New Phytol.

[b110] Thompson AR (2005). Dynamics of demographically open mutualists: immigration, intraspecific competition, and predation impact goby populations. Oecologia.

[b111] Tuomisto H (2011). Commentary: do we have a consistent terminology for species diversity? Yes, if we choose to use it. Oecologia.

[b112] Urban MC, Leibold MA, Amarasekare P, Gomulkiewicz L, De Meester R, Hochberg ME (2008). The evolutionary ecology of metacommunities. Trends Ecol. Evol.

[b113] Vacharaksa A, Finlay BB (2010). Gut microbiota: metagenomics to study complex ecology. Curr. Biol.: CB.

[b114] Violle C, Navas M-L, Vile D, Kazakou E, Fortunel C, Hummel I (2007). Let the concept of trait be functional!. Oikos.

[b115] Webb CO, Ackerly DD, McPeek MA, Donoghue MJ (2002). Phylogenies and community ecology. Annu. Rev. Ecol. Evol. Syst.

[b116] Webb CO, Losos JB, Agrawal AA (2006). Integrating phylogenies into community ecology. Ecology.

[b117] Weitz JS, Poisot T, Meyer JR, Flores CO, Valverde S, Sullivan MB (2013). Phage–bacteria infection networks. Trends Microbiol.

[b118] Wetzel CE, Bicudo DdeC, Ector L, Lobo EA, Soininen J, Landeiro VL (2012). Distance decay of similarity in neotropical diatom communities. PLoS ONE.

[b119] Whitham TG, Bailey JK, Schweitzer JA, Shuster SM, Bangert RK, LeRoy CJ (2006). A framework for community and ecosystem genetics: from genes to ecosystems. Nature rev. Genet.

[b120] Xie W, Meng Q-S, Wu Q-J, Wang S-L, Yang X, Yang N-N (2012). Pyrosequencing the Bemisia tabaci Transcriptome Reveals a Highly Diverse Bacterial Community and a Robust System for Insecticide Resistance. PLoS ONE.

[b121] Yang Z, Rannala B (2012). Molecular phylogenetics: principles and practice. Nat. Rev. Genet.

[b122] Yu DW, Ji Y, Emerson BC, Wang X, Ye C, Yang C (2012). Biodiversity soup: metabarcoding of arthropods for rapid biodiversity assessment and biomonitoring. Methods Ecol. Evol.

[b123] Zhang L, Thygesen UH, Knudsen K, Andersen KH (2012). Trait diversity promotes stability of community dynamics. Theoretical Ecology.

